# Louisiana Medicaid access for treatment and care for hepatitis C virus (LA-MATCH) project: A cross-sectional study protocol

**DOI:** 10.1371/journal.pone.0257437

**Published:** 2021-10-06

**Authors:** Hasheemah Afaneh, Susanne Straif-Bourgeois, Evrim Oral, Ashley Wennerstrom, Olivia Sugarman, William T. Robinson, Angel Whittington, Edward Trapido

**Affiliations:** 1 Department of Epidemiology, New Orleans School of Public Health, Louisiana State University Health Sciences Center, New Orleans, Louisiana, United States of America; 2 Department of Biostatistics, New Orleans School of Public Health, Louisiana State University Health Sciences Center, New Orleans, Louisiana, United States of America; 3 Department of Behavioral and Community Health Sciences and Center for Healthcare Value & Equity, New Orleans School of Public Health, LSU Health Sciences Center–New Orleans, Louisiana State University Health Sciences Center, New Orleans, Louisiana, United States of America; 4 University of Louisiana at Monroe, Medicaid, Monroe, Lousiana, United States of America; University of New Mexico Health Sciences Center, UNITED STATES

## Abstract

**Introduction:**

This article presents the Louisiana Hepatitis C Elimination Program’s evaluation protocol underway at the Louisiana State University Health Sciences Center–New Orleans. With the availability of direct-acting antiviral (DAA) agents, the elimination of Hepatitis C (HCV) has become a possibility. The HCV Elimination Program was initiated by the Louisiana Department of Health (LDH) Office of Public Health (OPH), LDH Bureau of Health Services Financing (Medicaid), and the Louisiana Department of Public Safety and Corrections (DPSC) to provide HCV treatment through an innovative pricing arrangement with Asegua Therapeutics, whereby a fixed cost is set for a supply of treatment over five years.

**Materials and methods:**

A cross-sectional study design will be used. Data will be gathered from two sources: 1) an online survey administered via REDCap to a sample of Medicaid members who are receiving HCV treatment, and 2) a de-identified data set that includes both Medicaid claims data and OPH surveillance data procured via a Data Use Agreement between LSUHSC-NO and Louisiana Medicaid.

**Discussion:**

The evaluation will contribute to an understanding of the scope and reach of this innovative treatment model, and as a result, an understanding of areas for improvement. Further, this evaluation may provide insight for other states considering similar contracting mechanisms and programs.

## Introduction

About 75%–85% of people infected with Hepatitis C virus (HCV) will become chronically infected, resulting in long-term health issues, such as liver cancer, or even, death [1,2]. The Louisiana Department of Health (LDH) estimates that about 50,000 Louisiana residents live with chronic HCV infection [3]. Data from 2013–2016 indicate that the infection rate for HCV in Louisiana is 1,420 per 100,000, which is higher than the national rate at 926 per 100,000 [4]. Furthermore, due to the high cost of the twelve-week HCV treatment regimen, less than 3% of persons infected with HCV and enrolled in Medicaid received treatment in 2017 [3]. With twenty percent of those diagnosed with HCV living in poverty and a median household income of under $45,374 [5], the cost of the therapy regimen is a barrier to receiving treatment.

The HCV Elimination Program (Program) was initiated by the Louisiana Department of Health (LDH) Office of Public Health (OPH), LDH Bureau of Health Services Financing (Medicaid), and the Louisiana Department of Public Safety and Corrections (DPSC) in June 2019 [3]. The goal is to provide HCV treatment [6,7] to all Louisiana Medicaid members and people in DPSC custody who are living with HCV. The Program uses an innovative pricing arrangement, referred to as the “subscription model" [8–10]. For HCV treatment, LDH and a pharmaceutical company have negotiated a five-year agreement, whereby Medicaid and the DOC can pay for a certain number of the treatment regimen with sofosbuvir/velpatasvir (Sofvel/Epclusa) for no more than a fixed cost [10–12]. In turn, the treatment is offered to the Medicaid and DPSC populations living with HCV at no cost. Sofosbuvir/velpatasvir is a direct-acting antiviral (DAA) agent in a single tablet prescribed once per day over 12 weeks; it has been deemed a safe and effective treatment for HCV and achieves almost 95% sustained virologic response (SVR) [11,12].

The Program’s goals are to cure current chronic HCV infections and prevent potentially serious health problems and deaths, with longer term aspiration to significantly reduce or eliminate ongoing transmission of HCV among Louisiana residents [8]. The goal of our evaluation, the Louisiana Medicaid Access Treatment and Care for HCV (LA-MATCH) Project, is to better understand the scope of reach, impact, and uptake of curative HCV treatment among Medicaid members before and after the Medicaid contract and public health activities were in place.

## Materials and methods

The evaluation is occurring between November 2019 through June 2021. Survey data will be collected from November 2019 –June 2021. Restricted datasets from Medicaid will include data from January 1, 2016 –present. This evaluation is approved by both the Louisiana State University Health Sciences Center—New Orleans (LSUHSC-NO) and the Louisiana Department of Health (LDH) Institutional Review Boards (IRBs) as exempt.

### Aims

We will complete this evaluation through the following aims. DPSC activities were not included in this evaluation due to funding constraints.

The first aim of this evaluation is to analyze the numbers, trends, and characteristics of people living with HCV covered by Medicaid and prescribed sofosbuvir/velpatasvir under the prescription program. We hypothesize that more than 95% of individuals who test positive for HCV after the start of the elimination program will have a sofosbuvir/velpatasvir prescription within the Medicaid claims database. We will also determine if individuals who take multiple medications are more likely to fill and refill their sofosbuvir/velpatasvir prescription than those who take no other medication or one medication. We hypothesize that individuals accustomed to taking multiple medications will be more likely to fill and refill their sofosbuvir/velpatasvir prescriptions than individuals who take no other or one medication.

The second aim is to analyze and compare the characteristics of individuals who are newly diagnosed with HCV who fill their prescriptions for sofosbuvir/velpatasvir compared to individuals who are newly diagnosed with HCV who do not fill their prescriptions. To complete this aim, we will:

- Determine if individuals diagnosed with HCV between July 15, 2019 and February 2021 are more likely to fill their prescriptions than those diagnosed before July 15, 2019. We hypothesize that the individuals who were diagnosed with HCV between July 15, 2019 and February 2021 were more likely to fill their prescriptions than those diagnosed before July 15, 2019. Additionally, we will evaluate if Covid-19 pandemic caused interruption in their treatment or caused any challenges using the survey data.
- Evaluate whether individuals born between 1945 and 1965, which compromise the highest incidence of chronic HCV infections [13], adhere more in their sofosbuvir/velpatasvir use than younger individuals. We hypothesize that older individuals who have HCV may have had their infection longer, and thus, may have been waiting for definitive treatment that is affordable than younger individuals. Therefore, older individuals may be more likely to be adherent than younger people.
- Establish whether people who inject drugs (PWIDs) are more adherent with sofosbuvir/velpatasvir prescriptions than those who do not report injection drug use. We hypothesize that PWIDs may fill and refill their prescriptions more consistently than those who are not current PWIDs.
- Ascertain whether individuals referred from syringe service programs (SSPs) are more adherent to sofosbuvir/velpatasvir treatment than those referred from other programs and medical practices. We hypothesize that individuals referred from SSPs will be more adherent to treatment than those referred from other programs and medical practices.

The third aim is to analyze data on medical providers and facilities (i.e. medical clinics, community-based clinics, and hospitals) that prescribe sofosbuvir/velpatasvir under this program. We hypothesize that there was a growing number of sofosbuvir/velpatasvir prescriptions through March 2020, and then, due to COVID-19, the number of prescriptions may have declined because the number of individuals being treated for HCV declined. To complete this aim, we will analyze the type by specialty and LDH region and facility type of provider prescribing sofosbuvir/velpatasvir under the Program, and compare prescription rates to the completion of HCV treatment to determine the types of providers and facilities are most likely to participate in the program and have participants complete treatment. The success of HCV treatment will be measured by a prescription claim and a negative lab result (HCV viral load) after 3 months. We hypothesize that providers in urban areas were earlier to prescribe HCV treatment under this Program than those in rural areas. We will also determine if sofosbuvir/velpatasvir recipients are being written prescriptions primarily by providers specialty (e.g. internal medicine, GI) or at various types of facilities (e.g. FQHCs, hospitals) and assess the completion rates stratified by the type of provider and facility. We hypothesize that facilities that serve a large population of Medicaid insured individuals will have the largest number of prescriptions.

### Study design

We will use a cross-sectional study design with two data sources: 1) an online survey administered via REDCap to a sample of participants who are eligible to benefit from the new subscription program (i.e. those who are Medicaid members) and 2) a de-identified data set that includes both Medicaid claims data and the Office of Public Health surveillance data, which is procured via a Data Use Agreement between LSUHSC-NO and Medicaid.

## Methods and analysis

To achieve Aim 1, the evaluation team will obtain restricted datasets from Medicaid, including pharmacy and encounter information. We will use datasets from 2016 forward. Our inclusion criteria for the dataset include: a) two medical services with a diagnosis code of hepatitis C on or after January 1, 2016 (excluding diagnosis codes attached to lab, transportation, and pharmacy claim types) and b) any DAA prescription dispensed to the member on or after January 1, 2016. All statistical analyses will be carried out using SAS version 9.4, and all tests will be carried out at the 5% confidence level. Benjamini-Hochberg procedure will be applied for multiple comparisons to control for the false discovery rate at level 0.05. We will calculate descriptive statistics (e.g., means, medians, ranges, standard deviations, or frequencies as appropriate) to estimate the variables’ distributional properties. We will compare differences in variables before and after sofosbuvir/velpatasvir treatment using two-sample t-tests for continuous variables and using z- or Pearson chi-square test statistics for binary/categorical variables. Similarly, we will compare differences between individuals who were prescribed sofosbuvir/velpatasvir with those who were living with HCV and not prescribed medication using two-sample t-tests, z-tests, or Pearson chi-square test statistics depending on the nature of the variables. We will also assess the differences in variables for race, gender, and age groups using two-sample t-tests, ANOVA, or Pearson chi-square test statistics, depending on the nature of the variables. We will evaluate if persons who take multiple medications are more likely to fill and refill their sofosbuvir/velpatasvir than those who take one or no other medications using logistic regression modeling.

To achieve Aim 2, we constructed a survey tool described in detail below.

### Survey participants

The eligibility criteria for the survey are: a) 18 years of age or older; b) enrolled in Louisiana Medicaid at time of the survey; c) currently a Louisiana resident; d) currently prescribed sofosbuvir/velpatasvir for HCV, and e) diagnosed with HCV. Potential eligible participants will be identified through the following agencies: physicians’ offices; Federally Qualified Health Clinics (FQHCs); clinics; medical centers; primary care centers; hospitals; methadone clinics; buprenorphine providers; and SSPs.

### Sampling procedure

**Fig 1:**
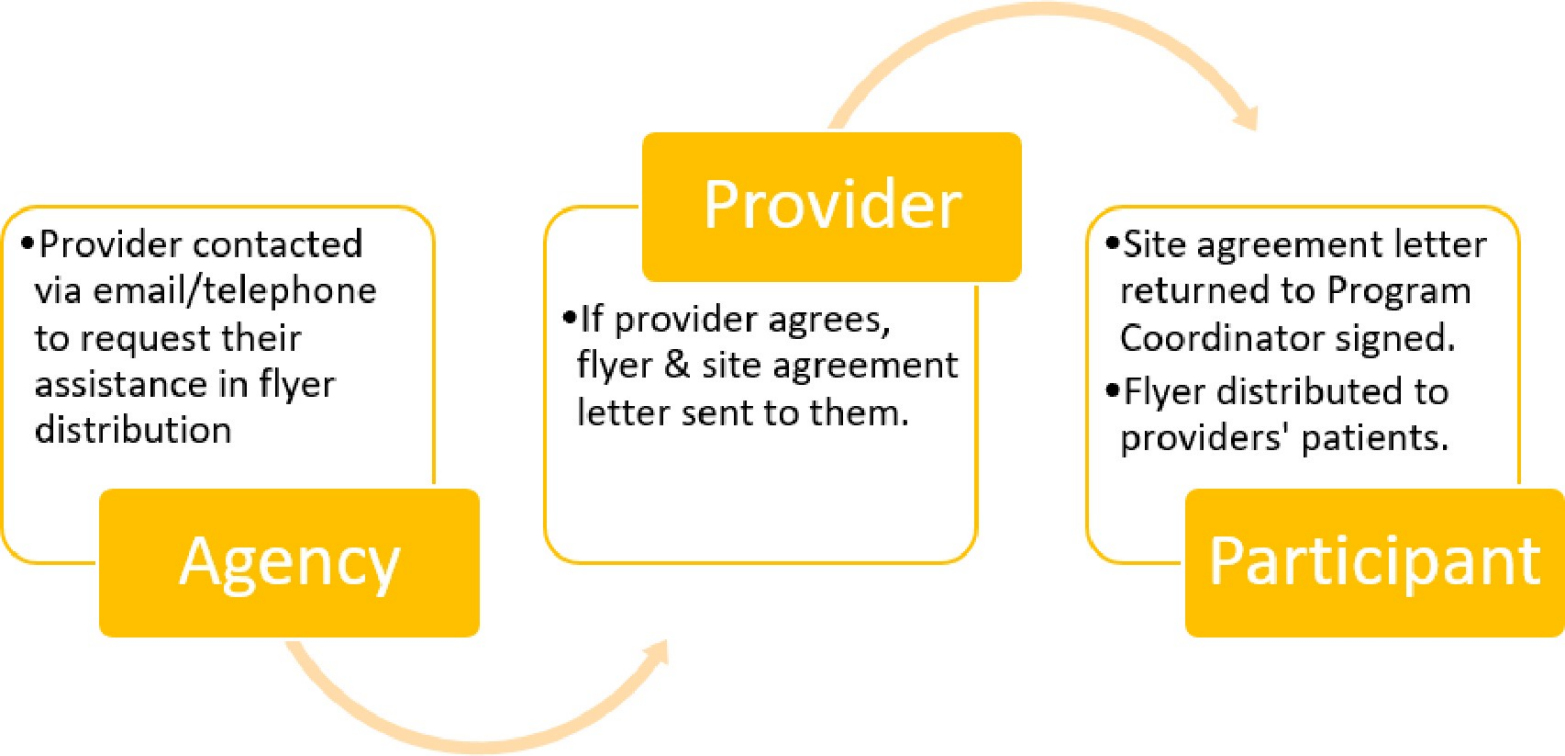
Agency/Provider outreach for participant recruitment for Part 1 Aim 2.

We will sample 200 HCV positive individuals who are prescribed sofosbuvir/velpatasvir and covered by who are Medicaid members from July 2019 and onward. We will use a two-stage convenience sampling approach: The first stage will use simple random sampling to select 40 agencies from a publicly available list provided by LDH-OPH of 131 agencies with medical providers who prescribe sofosbuvir/velpatasvir and accept Medicaid as a form of insurance. After randomly selecting 40 agencies (primary sampling unit), we will contact the providers at the selected agencies via email or phone call to arrange for an LA-MATCH flyer to be given to their clients a prescription for sofosbuvir/velpatasvir and on Medicaid. Emails and phone calls will follow scripts that introduce the LA-MATCH Project and describe the survey’s purpose. We will ask providers to post flyers in their clinics and describe the study and distribute flyers to potentially eligible patients. If the provider refuses, their refusal will be documented in an Excel spreadsheet along with the reason (if any). If providers indicate verbally that they are willing to participate, we will send via a site support letter and copies of the flyers via email or mail. Signed site support letters will be submitted to the LSUHSC-NO IRB for the record. After a provider agrees to participate, we will request that they send the survey flyer to all eligible patients (i.e. through email, hanging on the wall, handing out in waiting room) (second stage). The flyer will provide information on how to complete the survey online or by phone. The second stage is convenience sampling; we will survey the first five participants that contact us from each selected agency. Fig 1 below provides a brief overview of this outreach.

### Survey

We will utilize a concurrent mixed-mode survey design to increase the participation rate. Participants will have the option to do the survey either via the web or by phone. The web survey, which is a total of 54 questions, will be administered through REDCap [14], an online data capture program to collect information on the patient’s risk behaviors, HCV knowledge, facilitators, and barriers to treatment. Specifically, the survey will ask questions in the following domains: general HCV information (i.e. treatment, side effects); health behaviors (i.e. needle use and alcohol and tobacco use); psychosocial (i.e. quality of life); barriers to care (i.e. social determinants of health); and demographics. Only IRB-approved study staff will answer the phones and complete the consent, eligibility screening, and survey process with participants should the participant choose to complete the survey by phone. The entirety of the consent form will be available in written form on REDCap. Participants will indicate consent using a check box in REDCap. Whether through the phone or online, the first task after consenting to take the survey will be to confirm eligibility by answering a few questions. All potential participants will be informed that participation in the LA-MATCH project evaluation will not affect their ability to be treated. Participants will also be notified prior to consent that there is a monetary compensation of $10 and a facemask for completing the survey. Data collection will continue until May 20, 2021.

### Medicaid and OPH data

All information will be requested from the Louisiana Office of Medicaid. OPH will provide data from the HCV surveillance data, and this data will be matched with the Medicaid datasets. The matched dataset sent from OPH to Medicaid will not include SSN and other identifiers. The purpose of matching is to determine comparative program efficacy (determined by seropositivity and viral load from laboratory data) by participant characteristics, behaviors, and perceptions, as reported in the survey. Furthermore, Medicaid claims data will have the information on whether the sofosbuvir/velpatasvir prescriptions were filled. Sex, age, date of HCV diagnosis, date of enrollment into Medicaid, healthcare provider information from the Medicaid enrollment database, and provider claims database, will also be collected. Some risk factor information will be obtained from the subset of individuals who complete the survey. Data will also be obtained from Medicaid claims data and OPH surveillance data. We will look at risk factors, comorbidities, issues with distance from home to the providers (from survey responders), laboratories, and dispensing pharmacy.

We will calculate descriptive statistics to estimate the distributional properties of the variables from the survey tool. As a first choice, parametric methods will be used (Box-Cox transformations will be applied to skewed variables); however, we will also consider nonparametric or robust alternatives depending on the nature of the data and statistical model assumptions. Summaries and comparisons of characteristics of patients who fill sofosbuvir/velpatasvir prescriptions with respect to patients who do not fill sofosbuvir/velpatasvir prescriptions will be performed using chi-square paired t-tests, or Wilcoxon signed rank tests. We will assess if persons diagnosed with HCV after July 15, 2019 were more likely to fill their prescriptions than those diagnosed before July 15, 2019 using logistic regression modeling. We will evaluate whether persons born between 1945 and 1965 are more adherent in their sofosbuvir/velpatasvir use than younger patients using the z- or chi-square test. We will also compare adherence in sofosbuvir/velpatasvir use between current IV drug users and non-IV drug users using z- or chi-square test statistic. Similarly, to assess whether persons referred from needle exchange programs are more adherent with their sofosbuvir/velpatasvir use with respect to those from other medical practices, we will utilize z- or chi-square test statistic. Finally, we will assess potential risk factors (such as age, race, gender, sexual orientation, and health behaviors) affecting non-adherence in sofosbuvir/velpatasvir use via a multivariable logistic regression model.

To achieve Specific Aim 3, provider and agency information will be obtained from Medicaid claims data. Demographic data of prescribers will be obtained from LA Medicaid enrollment data and survey data. Sofosbuvir/velpatasvir prescriptions will come from pharmacy data. We will calculate means, medians, ranges, standard deviations, or frequencies of the variables stratified by the location of providers prescribing HCV treatment under the Program. Similarly, we will calculate descriptive statistics on variables stratified by provider training type and facility type separately. We will assess differences for provider locations, provider training types, or facility types using ANOVA or Pearson chi-square test statistics, depending on the nature of the variables. We will evaluate the differences between provider types or facility types with respect to the demographic subgroups using Pearson chi-square test statistics. Multiple logistic regression will explore determinants of successful treatment using potential factors, such as patient demographics, patient health behaviors, agency type, and facility size (i.e., larger provider vs. small provider).

We performed a power analysis using PASS software for aims 1 and 2 separately. For aim 1, we assumed that medication adherence would be around 60% among HCV patients [15,16]. Since we expect to have approximately 50,000 patients from Medicaid data, we have enough power for planned logistic regression modeling for aim 1 (Table 1). For aim 2, we assumed that current persons who inject drugs (PWIDs) are more compliant with sofosbuvir/velpatasvir prescriptions than non-IV drug users. As can be seen from Table 1, a total sample size of 200 achieves 80% power to detect a difference between the two group proportions of 0.18 at 0.05 significance level (the proportion of adherence among non-IV drug users were assumed to be 60% in the calculations).

**Table 1:** Power analyses at 0.05 significance level.

**Specific Aim 1** Necessary minimum sample sizes needed to achieve 80% power for various odds ratios at 0.05 significance level										
Odds Ratio:	1.2	1.3	1.4	1.5						
Min sample size needed:	994	486	299	209						
**Specific Aim 2** Power values for n = 200 for various group proportion differences at 0.05 significance level										
Difference in group proportions:	0.10	0.15	0.16	0.17	0.18	0.19	0.20	0.25	0.30	0.35
Power:	0.32	0.63	0.69	0.75	0.80	0.85	0.89	0.98	1.00	1.00

## Discussion

Limitations of this study protocol include the cross-sectional design and COVID19 that limits in-person participant recruitment to take the survey due to overload of provider workloads as well as physical distancing measures. Furthermore, our sampling methods might introduce selection bias. We plan to utilize appropriate weighting procedures to reduce the possible effects of selection bias on our estimates. The evaluation team plans to disseminate these results through written reports to the LDH Office of Public Health and Office of Medicaid. If any amendments to the study are needed, the IRB will be notified and an amendment will be submitted with the necessary changes for their review. The Louisiana Medicaid Access for Treatment and Care for HCV (LA-MATCH) Project can reduce the burden of cost by providing free access to treatment to those living with HCV, enrolled in Medicaid in Louisiana, and currently incarcerated by DPSC. Furthermore, this is one of two programs [17] currently following the Netflix model in the United States, and as such, if successful, has the potential to serve as a model for replication in other states that aim to reduce the burden of HCV in high-risk populations. The evaluation aims to provide an understanding who is enrolled in the elimination program, fill their prescriptions, and complying with the treatment regimen, and as a result, be able to identify areas for increased enrollment and improvement in the elimination program.

## References

[1] Centers for Disease Control and Prevention. Hep C Questions and Answers for the Public [Internet]. cdc.gov.; Published 2018; cited 2020 Sept. 06. Available from: https://www.cdc.gov/hepatitis/hcv/cfaq.htm#statistics.

[2] Centers for Disease Control an. Hep C Questions and Answers for Health Professionals: Transmission and Symptoms [Internet] cdc.gov; Published 2018; cited 2020 Sept. 06. Available from https://www.cdc.gov/hepatitis/hcv/hcvfaq.htm#section1.

[3] Louisiana Department of Health. Louisiana launches hepatitis C innovative payment model with Asegua Therapeutics, aiming to eliminate the disease. *ldh*.*la*.*gov*. June 26, 2019.

[4] HepVu. Louisiana: Geographic Comparisons [Internet]; hepvu.org; N.d.; cited 2020 Sept. 10. Available from: https://hepvu.org/state/louisiana/#tab-geographic.

[5] HepVu. Louisiana: Other Comparisons [Internet]; hepvu.org; N.d.; cited 2020 Sept. 10. Available from: https://hepvu.org/state/louisiana/#tab-other.

[6] Yek C., de la Flor C., Marshall J. et al. Effectiveness of direct-acting antiviral therapy for hepatitis C in difficult-to-treat patients in a safety-net health system: a retrospective cohort study. *BMC Med* 15, 204 (2017). 10.1186/s12916-017-0969-3. PMC5694912 29151365

[7] Spengler U. Direct antiviral agents (DAAs)–A new age in the treatment of hepatitis C virus infection. *Pharmacol Ther*. 183; 118–126 (2018). doi: 10.1016/j.pharmthera.2017.10.009 29024739

[8] Office of the Governor. Louisiana launches Hep C innovative payment model with Asegua Therapeutics, aiming to eliminate the disease [Internet]. Gov.louisiana.gov; 2019 Jun 26; 2020 Oct 1. Available from: https://www.gov.louisiana.gov/index.cfm/newsroom/detail/2031.

[9] Trusheim M.; Cassidy W. and Bach P. Altnerative state-level financing for hepatitis C treatment–The ‘Netflix Model.’ *JAMA*. 320(19): 1977–1978 (2018). doi: 10.1001/jama.2018.15782 30383176

[10] Gee, R. Louisiana’s journey toward eliminating Hepatitis C [Internet]. Health Affairs Blog. Published 2019 Apr; 2020 Oct 1. Available from: https://www.healthaffairs.org/do/10.1377/hblog20190327.603623/full/.

[11] Anna LZ, Monia M. and Laura G. Sofosbuvir/Velpatasvir for the treatment of Hepatitis C virus infection. *Acta Biomed*. 89(3): 321–331 (2018). doi: 10.23750/abm.v89i3.7718 30333452 PMC6502110

[12] Asselah T., Marcellin P. and Schinazi R. Treatment of hepatitis C virus with direct-acting antiviral agents: 100% cure? *Liver Int*. 38 Suppl 1:7–13. doi: 10.1111/liv.13673 29427484 PMC7713514

[13] Schillie S, Wester C, Osborne M, Wesolowski L, Ryerson AB. CDC Recommendations for Hepatitis C Screening Among Adults–United States, 2020. MMWR Recomm Rep 2020; 69(No. RR-2): 1–17. doi: 10.15585/mmwr.rr6902a1 32271723 PMC7147910

[14] REDCap. https://www.project-redcap.org/.

[15] Cos T. A., Bartholomew T. S., & Huynh K. J. (2019). Role of behavioral health providers in treating hepatitis C. *Professional Psychology*: *Research and Practice*, 50(4), 246–254. doi: 10.1037/pro0000243

[16] Zhang S., Rust G., Cardarelli K., Felizzola J., Fransua M., & Stringer H. G. Jr. (2015). Adherence to highly active antiretroviral therapy impact on clinical and economic outcomes for Medicaid enrollees with human immunodeficiency virus and hepatitis C coinfection. *AIDS Care*, 27(7), 829–835. doi: 10.1080/09540121.2015.1021745 25814041 PMC4888046

[17] Washington State Health Care Authority. Washington approved to allow modified ‘subscription model’ for prescription drug payment in Apple Health (Medicaid). Published 2019 Jun 13. 2020 Nov 23. Available from: https://www.hca.wa.gov/about-hca/washington-approved-allow-modified-subscription-model-prescription-drug-payment-apple.

